# p53 plays a central role in the development of osteoporosis

**DOI:** 10.18632/aging.103271

**Published:** 2020-06-02

**Authors:** Tao Yu, Xiaomeng You, Haichao Zhou, Alex Kang, Wenbao He, Zihua Li, Bing Li, Jiang Xia, Hui Zhu, Youguang Zhao, Guangrong Yu, Yuan Xiong, Yunfeng Yang

**Affiliations:** 1 Department of Orthopedic Surgery, Tongji Hospital, Tongji University School of Medicine, Shanghai 200065, China; 2 Department of Orthopedic Surgery, Brigham and Women’s Hospital, Harvard Medical School, Boston, MA 02115, USA; 3 Center for Biomedical Engineering, Department of Medicine, Brigham and Women’s Hospital, Harvard Medical School, Cambridge, MA 02139, USA; 4 Department of Orthopaedics, Union Hospital, Tongji Medical College, Huazhong University of Science and Technology, Wuhan 430022, China

**Keywords:** osteoporosis, osteogenesis, bone mass density, p53

## Abstract

Osteoporosis is a metabolic disease affecting 40% of postmenopausal women. It is characterized by decreased bone mass per unit volume and increased risk of fracture. We investigated the molecular mechanism underlying osteoporosis by identifying the genes involved in its development. Osteoporosis-related genes were identified by analyzing RNA microarray data in the GEO database to detect genes differentially expressed in osteoporotic and healthy individuals. Enrichment and protein interaction analyses carried out to identify the hub genes among the deferentially expressed genes revealed *TP53*, *MAPK1*, *CASP3*, *CTNNB1*, *CCND1*, *NOTCH1*, *CDK1*, *IGF1*, *ERBB2*, *CYCS* to be the top 10 hub genes. In addition, p53 had the highest degree score in the protein-protein interaction network. In vivo and in vitro experiments showed that *TP53* gene expression and serum p53 levels were upregulated in osteoporotic patients and a mouse osteoporosis model. The elevated p53 levels were associated with decreases in bone mass, which could be partially reversed by knocking down p53. These findings suggest p53 may play a central role in the development of osteoporosis.

## INTRODUCTION

Osteoporosis is a metabolic disease characterized by decreased bone mass per unit volume, despite the bone tissue having normal calcification and a normal ratio of calcium salt and matrix [1]. Osteoporosis can occur in both genders at any age, but it is most common in postmenopausal women. Overall, the disease affects 30% of women and 12% of men at some point in their lifetimes [2], but it affects more than 40% of postmenopausal women [3]. Patients with osteoporosis usually have bone pain and are prone to having a fracture [1]. As one of the most commonly occurring chronic diseases among the elderly, osteoporosis has become a serious problem for public health care systems [4]. The main therapeutic strategies for treating osteoporosis are the use anti-resorptive and anabolic drugs. However, most of the drugs in these classes have limitations and adverse side effects [3].

The pathogenesis of osteoporosis has not yet been fully elucidated. Factors that inhibit osteogenesis, promote bone resorption, or cause bone microstructural destruction may play a role in the development of osteoporosis, and a variety of genes may be directly or indirectly involved. For example, mutations of *COLI* are responsible for osteogenesis imperfecta [5], while allelic variation of *TCIRGI* are significantly associated with low bone mineral density (BMD) in a certain population [6]. In addition, Xie et al. also suggested that *CTNNB1* and *TP53* may play crucial roles in primary osteoporosis [7]. Osteoporosis is thus potentially associated with multiple genes and may result from gene-environment interactions [2].

Bioinformatics technology has been used to integrate and analyze big data from public database repositories for several diseases. For instance, bioinformatic methods have demonstrated prevalent alterations in RNA methylation regulators across cancer types. It was concluded that the m^6^A regulators tightly correlate with the activation and inhibition of cancer pathways, and also correlate with prognostically relevant tumor subtypes [8].

In the present study, we applied similar bioinformatic analysis of an osteoporosis microarray dataset retrieved from the Gene Expression Omnibus (GEO) to explore the mechanism underlying osteoporosis. Identification and validation of differentially expressed genes (DEGs) suggest that p53 may play a key role in the development of osteoporosis.

## RESULTS

### Identification of DEGs

**Figure 1:**
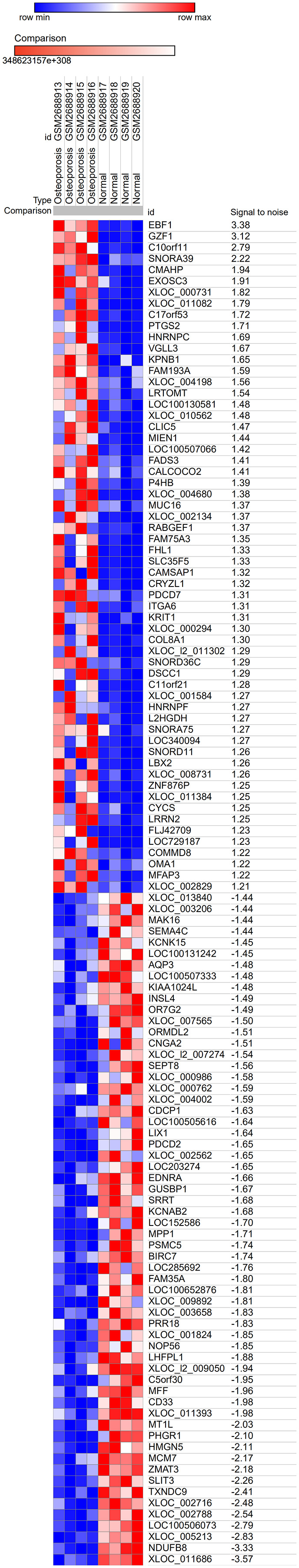
**Heat map of the top 60 DEGs in GSE100609 (30 upregulated and 30 downregulated).** The GSE100609 dataset, which included gene expression profiles from four healthy individuals and four osteoporotic patients, was obtained from the GEO database. In total, 228 upregulated and 281 downregulated DEGs were identified. Red, upregulation; blue, downregulation.

The GSE100609 dataset was obtained from the GEO database. It included gene expression profiles from 4 healthy individuals and 4 osteoporotic patients. Analysis of the dataset using the Morpheus online tool revealed 509 (228 upregulated and 281 downregulated) genes that were differentially expressed between the healthy group and the osteoporotic patients. The top 30 upregulated and downregulated genes are shown in Figure 1.

### Gene Ontology (GO) and Kyoto Encyclopedia of Genes and Genomes (KEGG) pathway enrichment analyses

**Figure 2:**
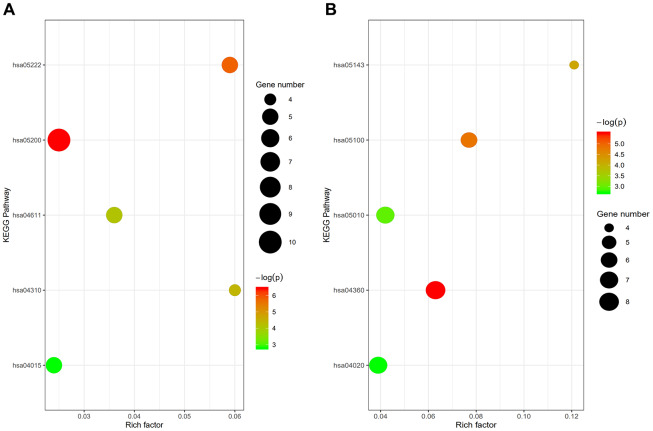
(**A**) Enrichment analysis of upregulated genes: hsa05200, cancer pathway; hsa05222, small cell lung cancer pathway; hsa04115, p53 signaling pathway; hsa04310, wnt signaling pathway; hsa04015, rap1 signaling pathway. (**B**) Enrichment analysis of downregulated genes: hsa04360, axon guidance pathway; hsa05100, bacterial invasion of epithelial cells pathway; hsa05143, African trypanosomiasis pathway; hsa05010, Alzheimer's disease pathway; hsa04020, calcium signaling pathway.

GO term analysis and KEGG pathway enrichment analyses were performed using the Database for Annotation, Visualization and Integrated Discovery (DAVID) bioinformatics tool. The results showed that under the biological processes category, upregulated DEGs in osteoporotic patients were significantly enriched in “Regulation of locomotion,” “Regulation of cellular component movement,” “Regulation of cell motility,” “Anatomical structure formation involved in morphogenesis,” and “Movement of cell or subcellular component.” On the other hand, the DEGs downregulated in osteoporotic patients were enriched in “Axon extension,” “Regulation of cellular component movement,” “Neuron projection extension,” “Developmental growth involved in morphogenesis,” and “Positive regulation of cellular protein metabolic process” (Table 1). The top five KEGG pathways for the DEGs upregulated in osteoporotic patients were “cancer pathway,” “small cell lung cancer pathway,” “p53 signaling pathway,” “Wnt signaling pathway,” and “rap1 signaling pathway.” The top five KEGG pathways for the DEGs downregulated in osteoporotic patients were “axon guidance pathway,” “bacterial invasion of epithelial cells pathway,” “African trypanosomiasis pathway,” “Alzheimer's disease pathway,” and “calcium signaling pathway” (Table 2 and Figure 2).

**Table 1:** GO analysis of DEGs involved in biological processes.

**Upregulated**			
**Term**	**Function**	**Count**	**P-value**
GO:0040012	Regulation of locomotion	15	1.5E-3
GO:0051270	Regulation of cellular component movement	15	2.2E-3
GO:2000145	Regulation of cell motility	14	2.9E-3
GO:0048646	Anatomical structure formation involved in morphogenesis	18	4.3E-3
GO:0006928	Movement of cell or subcellular component	24	5.3E-3
**Downregulated**			
	**Function**	**Count**	**P-value**
GO:0048675	Axon extension	8	2.7E-5
GO:0048588	Developmental cell growth	9	1.6E-4
GO:1990138	Neuron projection extension	8	1.7E-4
GO:0060560	Developmental growth involved in morphogenesis	9	3.0E-4
GO:0032270	Positive regulation of cellular protein metabolic process GO, gene ontology.	25	5.4E-4

**Table 2:** KEGG pathway analysis of DEGs.

**Upregulated**			
	Term*	**Count**	**P-value**
hsa05200	Cancer pathway	10	1.6E-3
hsa05222	Small cell lung cancer pathway	5	3.0E-3
hsa04115	p53 signaling pathway	4	1.2E-2
hsa04310	Wnt signaling pathway	5	1.6E-2
hsa04015	Rap1 signaling pathway	5	6.1E-2
**Downregulated**			
	Term*	**Count**	
hsa04360	Axon guidance pathway	8	4.1E-3
hsa05100	Bacterial invasion of epithelial cells pathway	6	8.0E-3
hsa05143	African trypanosomiasis pathway	4	1.5E-2
hsa05010	Alzheimer's disease pathway	7	5.2E-2
hsa04020	Calcium signaling pathway	7	6.7E-2

*The top 5 terms were selected based on P-values when more than five terms enriched terms were identified in a category.

### Protein-protein interaction (PPI) network construction and module analysis

**Figure 3:**
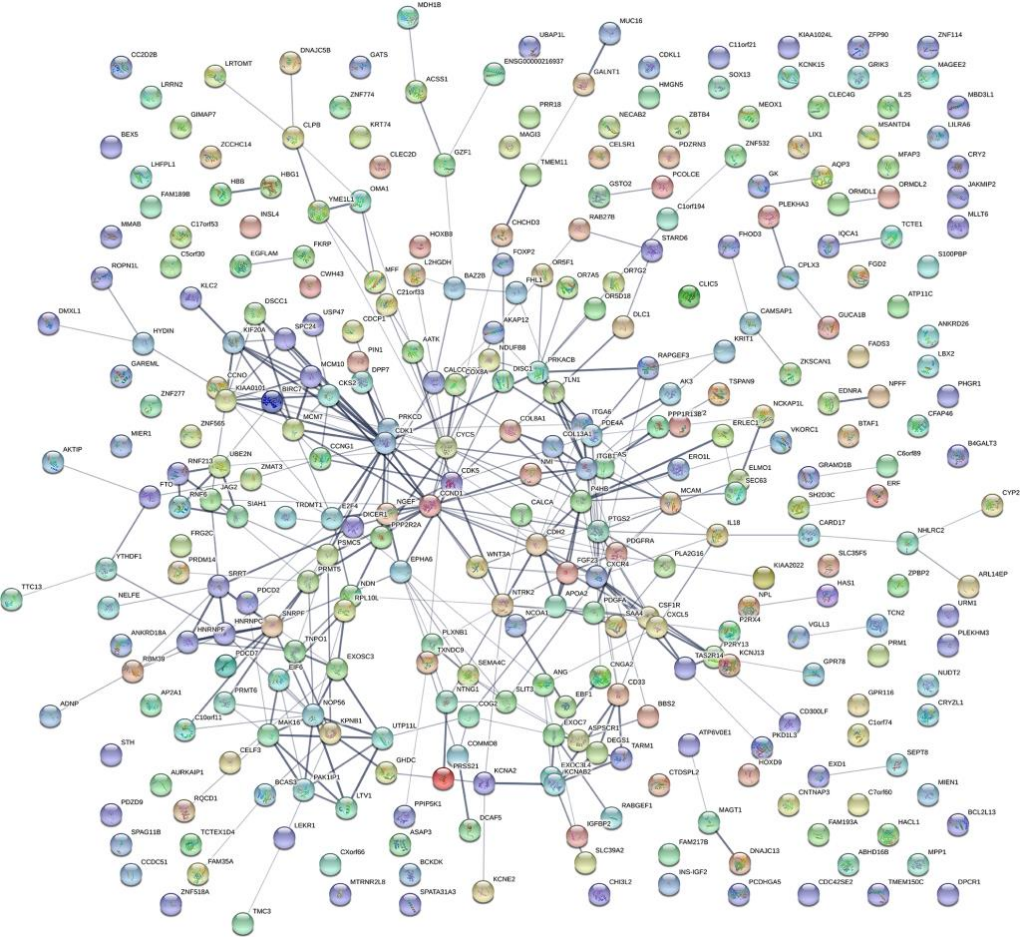
**Protein-protein interactions.** PPI network constructed using STRING and illustrating the interactions among DEGs.

**Figure 4:**
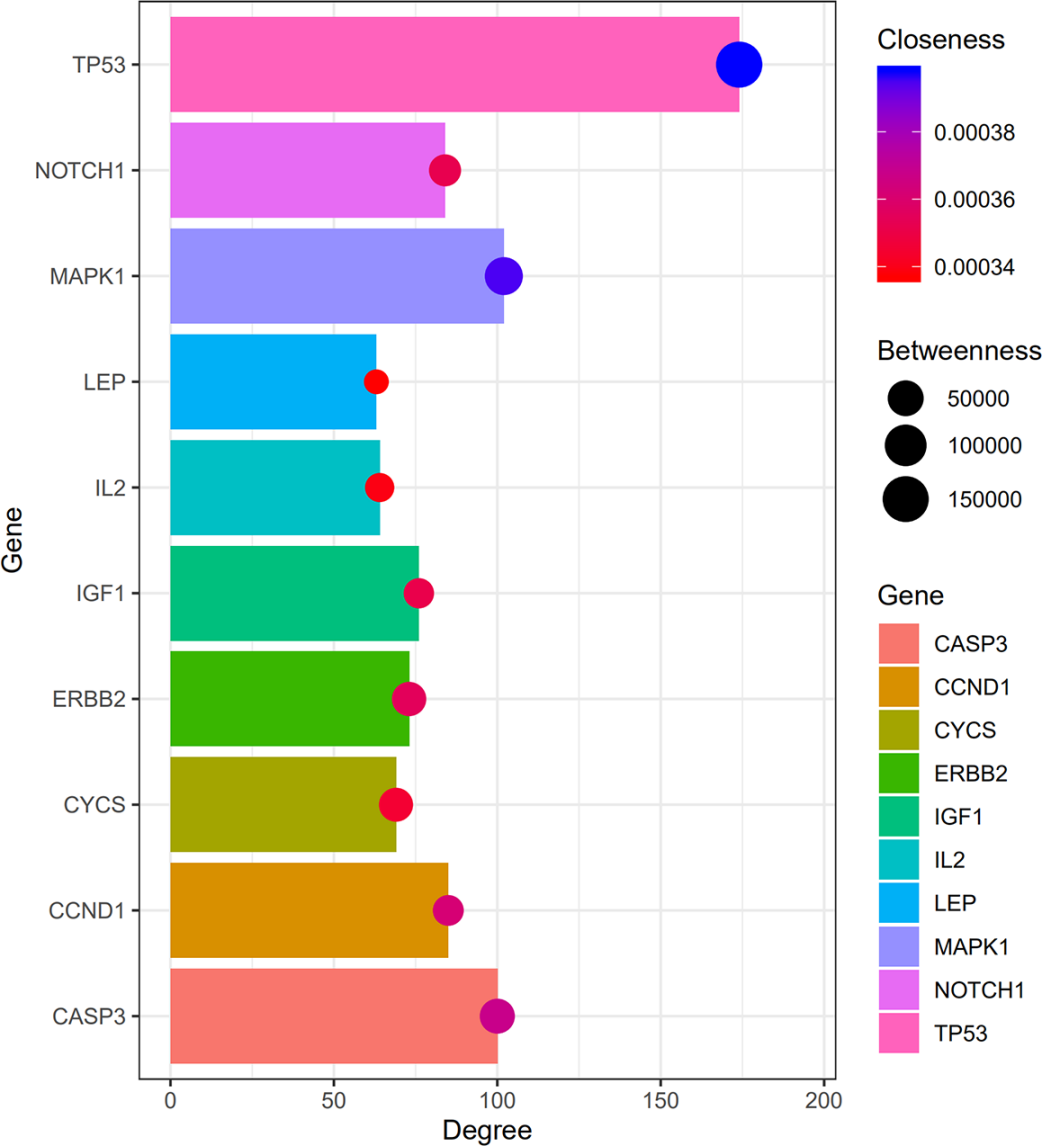
**Degree, betweenness, closeness of hub genes.** Within the PPI network, the top three hub genes with highest degree, betweenness and closeness scores are TP53, MAPK1, and CASP3.

A PPI network was constructed using STRING (Figure 3), and the top 10 hub genes with the highest degrees of interaction were determined with Cytoscape. These hub genes were *TP53*, *MAPK1*, *CASP3*, *CTNNB1*, *CCND1*, *NOTCH1*, *CDK1*, *IGF1*, *ERBB2*, and *CYCS* (Table 3). Gene centrality is represented by indexes of degree, closeness, and betweenness. Degree represented the degree of association of one node with all other nodes in the network. Closeness represented the closeness between a node and other nodes in the network. Betweenness was the number of times a node acted as the shortest bridge between two other nodes. The results for degree, closeness and betweenness are showed in Figure 4. Among the 10 hub genes, TP53 had the highest degree score of 174, indicating it potentially plays an important role during the development of osteoporosis.

**Table 3:** Degree scores for the top 10 upregulated genes.

**Gene ID**	**Gene name**	**Degree**
TP53	Tumor protein 53	174
MAPK1	Mitogen-Activated Protein Kinase 1	102
CASP3	Caspase 3	100
CTNNB1	Catenin, beta-1	89
CCND1	Cyclin D1	85
NOTCH1	notch receptor 1	84
CDK1	Cyclin-dependent kinase 1	81
IGF1	Insulin-like growth factor-1	76
ERBB2	erb-b2 receptor tyrosine kinase 2	73
CYCS	Somatic cytochrome C, somatic	69

**Figure 5:**
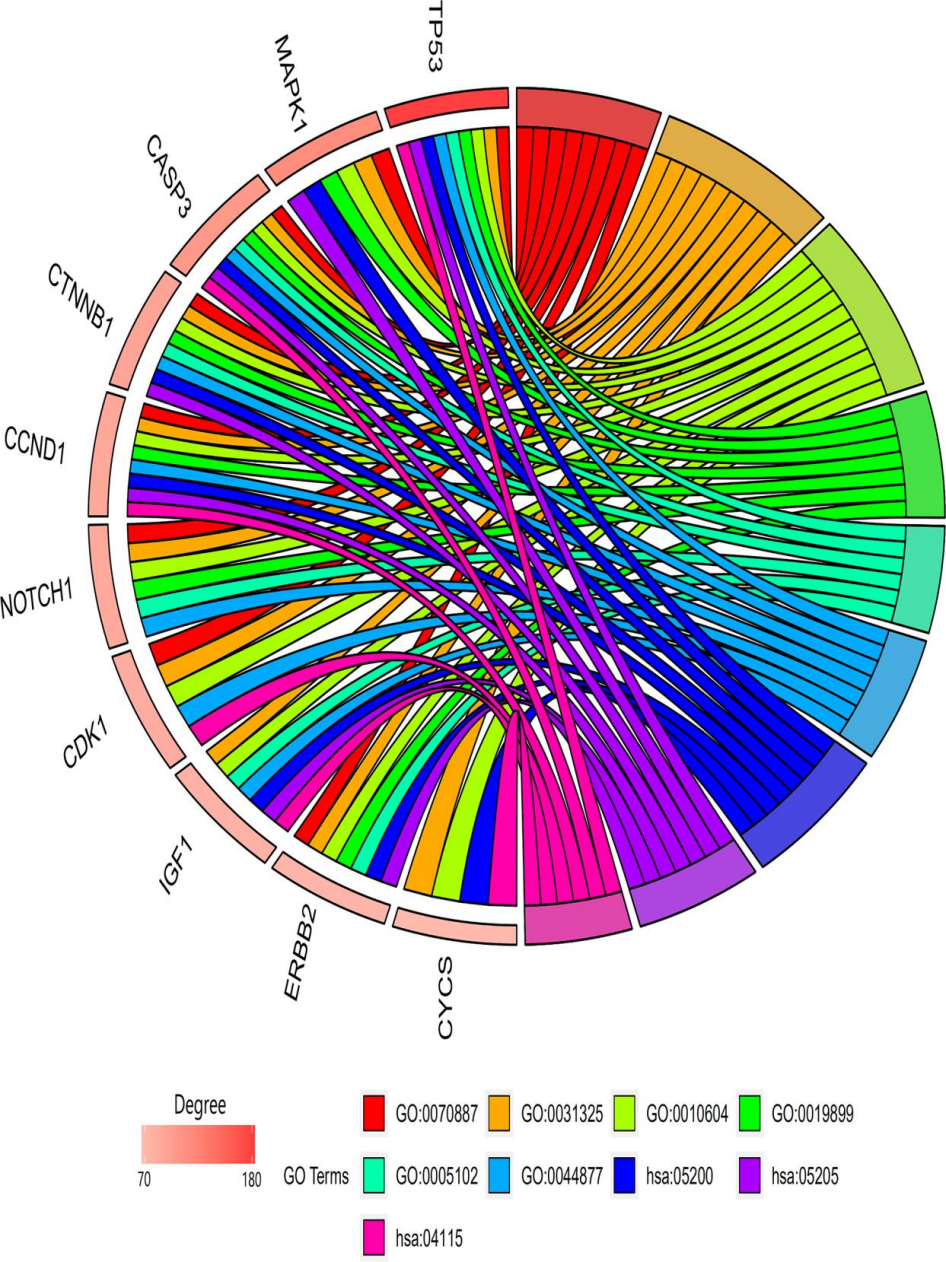
**GO terms/KEGG pathways associated with top 10 enriched hub genes.** Biological process module: GO:0070887, cellular response to chemical stimulus; GO:0031325, positive regulation of cellular metabolic process; GO:0010604, positive regulation of macromolecule metabolic process. Molecular functions module: GO:0019899, enzyme binding; GO:0005102, receptor binding; GO:0044877, macromolecular complex binding. KEGG pathway module: hsa05200, cancer pathway; hsa05205, proteoglycans in cancer pathway; hsa04115, p53 signaling pathway.

**Figure 6:**
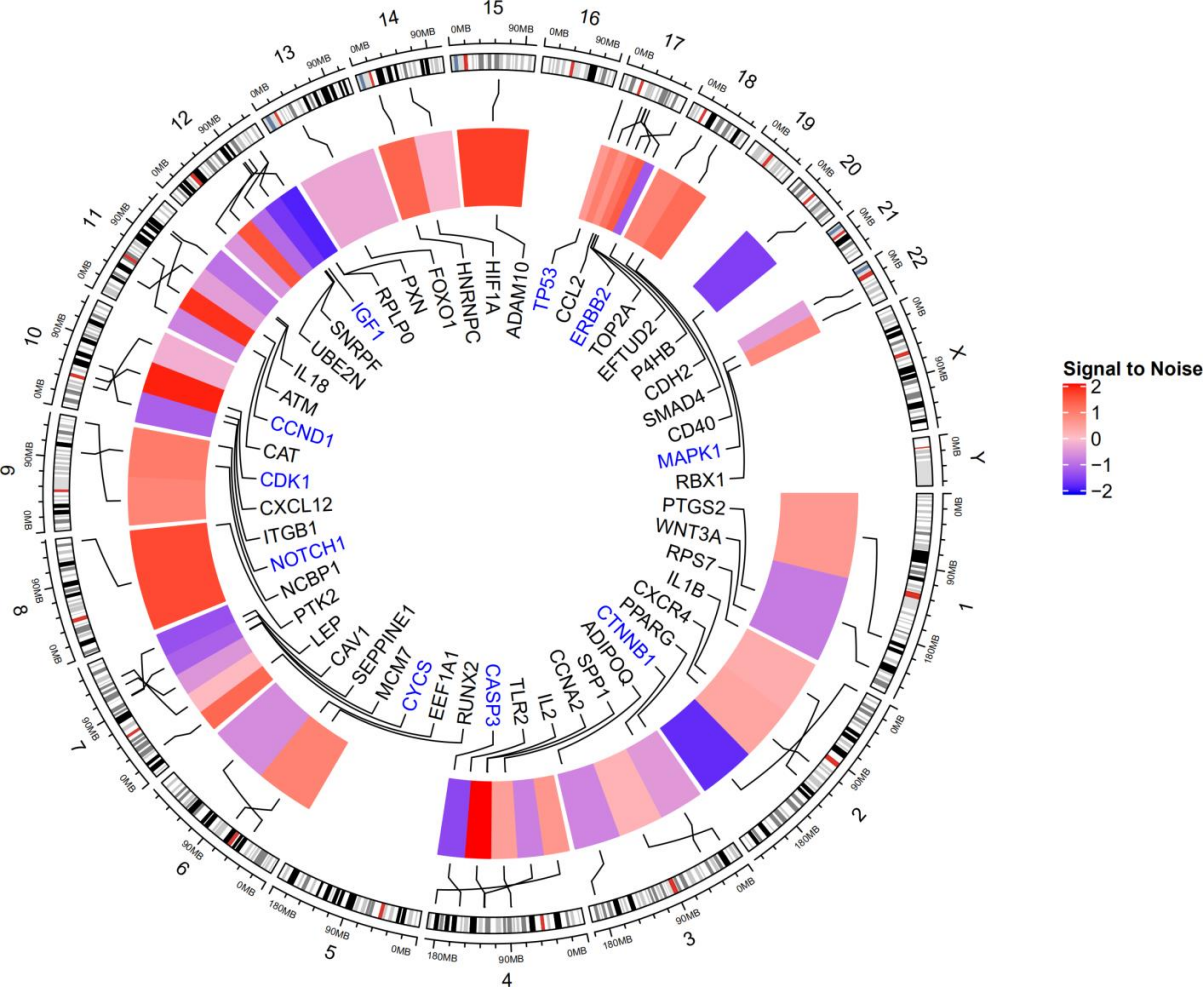
**Expression of top 50 genes based on degree score from the 6 modules and their positions on chromosome.** The hub genes *TP53*, *MAPK1*, *CASP3*, *CTNNB1*, *CCND1*, *NOTCH1*, *CDK1*, *IGF1*, *ERBB2*, and *CYCS* are highlighted in blue. *TP53*, *MAPK1*, and *CASP3* are located on chromosomes 17, 22, and 4, respectively.

The genes involved in the six PPI network modules with MCODE scores ≥5 and >5 nodes (Table 4) were characterized through GO term and KEGG pathway enrichment analyses. In the biological process category, the GO terms “Cellular response to chemical stimulus,” “Positive regulation of cellular metabolic process,” and “Positive regulation of macromolecule metabolic process” were significantly enriched. In addition, in the molecular function category, “Enzyme binding,” “Receptor binding,” and “Macromolecular complex binding” were also significantly enriched. Among KEGG pathways, “signaling pathway of cancer pathway,” “proteoglycans in cancer pathway,” and “p53 signaling pathway” were enriched (Table 5). In Figure 5, the top 10 genes associated with the enriched GO term/KEGG pathway are illustrated using a chord diagram. Expression of the top 50 genes from the 6 modules and their positions on chromosomes are also shown in Figure 6.

**Table 4:** Six modules from the PPI network with MCODE scores ≥5 and >5 nodes.

**Cluster**	**Score**	**Nodes**	**Edges**
1	17.15	69	583
2	10.39	47	239
3	7.00	7	21
4	5.75	49	138
5	5.625	17	45
6	5.00	5	10

**Table 5:** Functional and pathway enrichment analysis of the genes in each module.

**A, Biological process**			
Term*	**Name**	**Count**	**P-value**
GO:0070887	Cellular response to chemical stimulus	80	1.4E-18
GO:0031325	Positive regulation of cellular metabolic process	83	1.5E-18
GO:0010604	Positive regulation of macromolecule metabolic process	83	2.2E-18
**B, Molecular functions**			
Term*	**Name**	**Count**	**P-value**
GO:0019899	Enzyme binding	49	2.2E-9
GO:0005102	Receptor binding	43	4.2E-9
GO:0044877	Macromolecular complex binding	39	2.0E-8
**C, KEGG pathway**			
Term*	**Name**	**Count**	**P-value**
hsa05200	Cancer pathway	26	3.6E-8
hsa05205	Proteoglycans in cancer pathway	18	1.2E-7
hsa04115	P53 signaling pathway	10	3.2E-6

*The top 3 terms were selected based on P-values when more than 3 enriched terms were identified in a category.

### Downregulating p53 expression may protect against osteoporosis in vitro and in vivo

**Figure 7:**
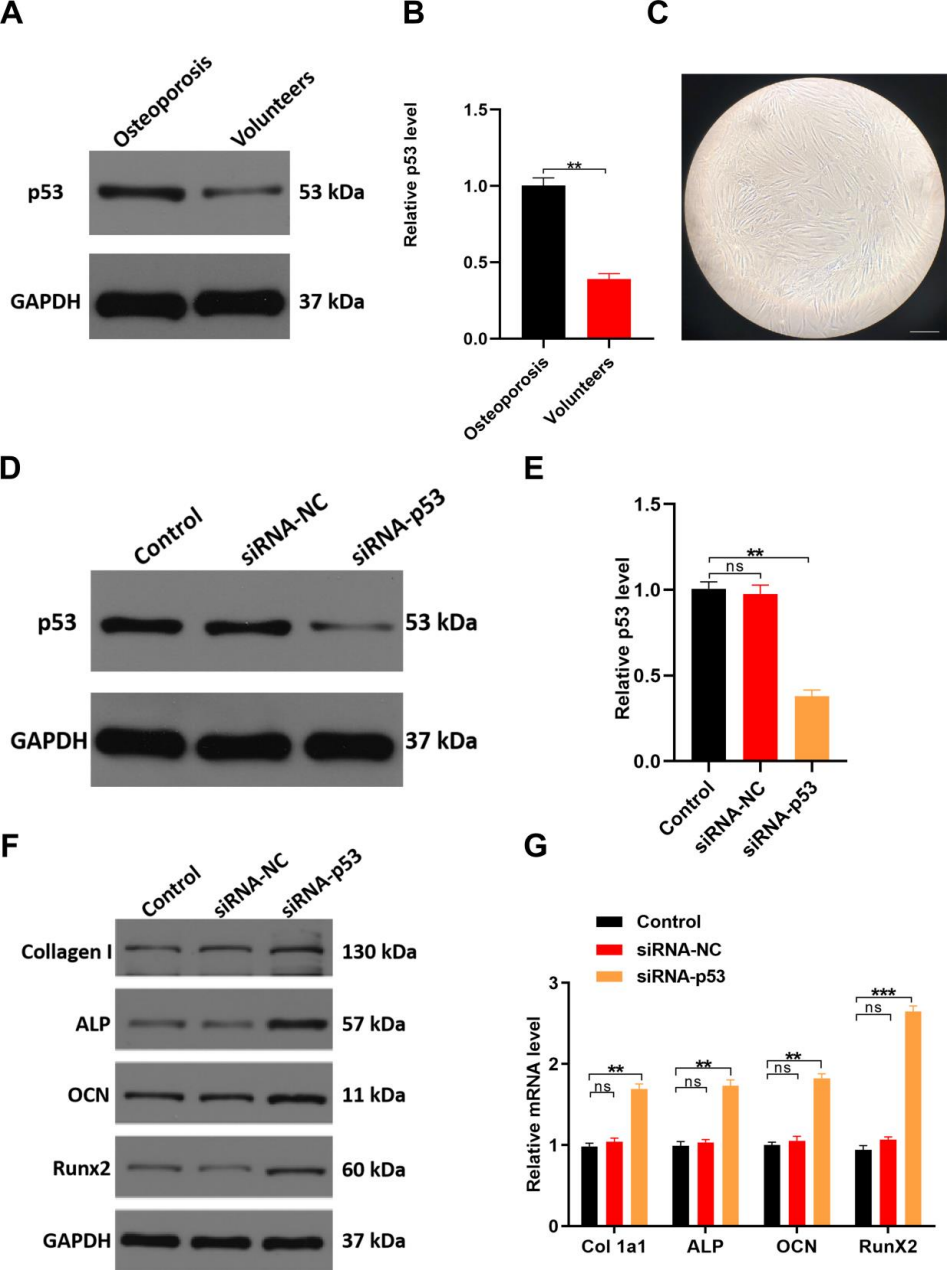
**Downregulating p53 expression may protect against osteoporosis in vitro.** (**A**, **B**) qRT-PCR and western blot analyses comparing p53 expression between healthy volunteers and osteoporosis patients. (**C**) Cellular morphology of hMSCs, scale bar, 50 μm. (**D**, **E**) hMSCs were treated with PBS, siRNA-NC, or siRNA-p53, after which p53 expression was assessed with qRT-PCR and western blotting. (**F**, **G**) qRT-PCR and western blot analysis of osteogenesis-related mRNAs in the three groups. Data are means±SD of triplicate experiments. *p < 0.05, **p < 0.01, ***p < 0.001.

Because *TP53* had the highest degree score among the top 10 hub genes, we hypothesized that p53 plays an important role during osteoporosis development. Consistent with our hypothesis, *TP53* gene expression was increased two-fold in the serum samples from osteoporotic patients compared to healthy controls (Figure 7B). Correspondingly, levels of p53 protein were also significantly increased in osteoporotic patients (Figure 7A), suggesting a potential role for p53 in osteoporotic development and/or progression.

In cultured human mesenchymal stem cells (hMSCs) transfected with siRNA targeting p53, expression levels of both p53 mRNA and protein were significantly decreased as compared to cells treated with PBS (control) or a negative control siRNA (siRNA-NC) (Figure 7D, 7E). As expected, expression levels of the osteogenesis-related genes Collagen Type I Alpha 1 (*Col 1a1*), Alkaline phosphatase (*ALP*), Osteocalcin (*OCN*) and RUNX Family Transcription Factor 2 (*RunX2*) were all significantly higher in p53 knockdown cells than in control (PBS) cells or cells expressing siRNA-NC (Figure 7F, 7G).

**Figure 8:**
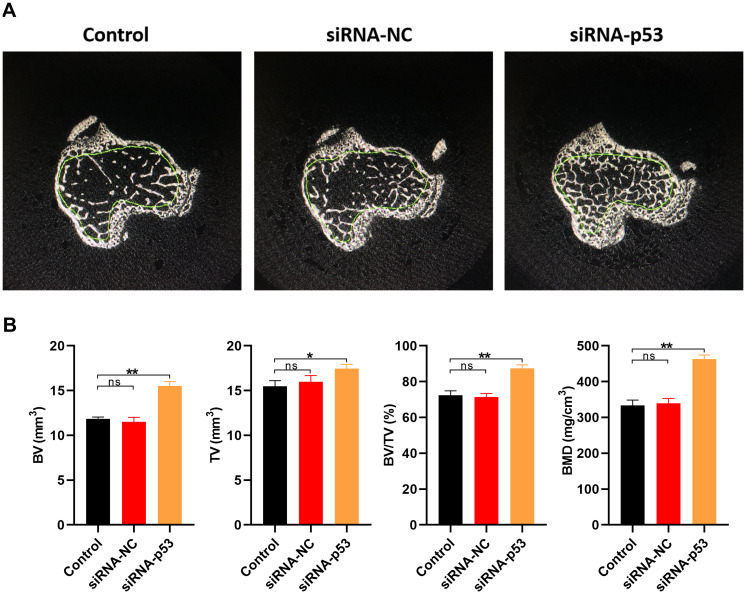
**Downregulating p53 expression may protect against osteoporosis in vivo.** (**A**) Cross sections of bone samples from osteoporosis model mice treated for 10 weeks with PBS, siRNA-NC, or siRNA-p53 (n=10 in each group). (**B**) BV, TV, BV/TV, and BMD values measured in osteoporosis model mice in each treatment group. Data are means±SD of triplicate experiments. *p < 0.05, **p < 0.01, ***p < 0.001.

The effect of p53 on osteoporosis was further investigated in vivo using a murine model. Osteoporosis was induced by oophorectomy the model mice treated with PBS (control), siRNA-p53 or siRNA-NC. Consistent with the in vitro experiments, bone volume (BV), total volume (TV), the BV/TV ratio and BMD were all significantly higher in the siRNA-p53 group than the control or siRNA-NC group (Figure 8). Thus, downregulating p53 appears to suppress features of osteoporosis both in vitro and in vivo.

## DISCUSSION

Osteoporosis is a metabolic disease characterized by low bone mass and microstructural destruction of bone tissue, resulting in increased bone fragility and proneness to fracture [1]. The pathogenesis of osteoporosis has not yet been fully elucidated. Factors that inhibit osteogenesis, promote bone resorption, and/or cause bone microstructural destruction can contribute to the development of osteoporosis.

The normal homeostasis of mature bone is maintained largely through the coordinated actions of regulating hormones and local cytokines [9]. Bone tissue continuously absorbs old bone and generates new bone, thereby maintaining a stable state of bone turnover [10]. With increasing age, however, the rate of bone turnover gradually declines, leading to gradual declines in BMD and bone mineral content (BMC) [11]. In general, the annual rate of BMD loss is about 0.5%, but the rate of BMD loss is usually slower in older men than women, in part because women experience a rapid loss of estrogen [12]. No such rapid hormonal change occurs in men [12]. The BMD loss is accompanied by distortion and destruction of bone microstructure. Some structures such as the trabeculae are unable to maintain normal morphology, resulting in narrowing, thinning, bending, and/or misalignment of trabecular bone, and even micro-injury or micro-fractures [13]. In some of the worse cases, the bone is completely absorbed and cavities develop. In those areas, the cortical bone becomes thinner, numbers of trabeculae decrease, and fragility increases until spontaneous compression fractures (e.g. in vertebral bodies) or transverse fractures (e.g. in the femoral neck or distal radius) occur [14].

Osteoporosis is a multifactorial disease [15] and a variety of genes and signaling pathways participate its pathogenesis [16]. In the present study, we analyzed the interactions among involved proteins and ranking the top 10 hub genes. The identified hub genes were *TP53*, *MAPK1*, *CASP3*, *CTNNB1*, *CCND1*, *NOTCH1*, *CDK1*, *IGF1*, *ERBB2*, and *CYCS*. Moreover, we found that nearly all of the top 10 hub genes were involved in the top five enriched GO terms or KEGG pathways, indicating their potential roles in osteoporosis progression. Consistent with our findings, a number of studies have previously reported the involvement of *TP53*, *MAPK1*, *CASP3*, *CTNNB1*, *CCND1*, *NOTCH1*, *CDK1*, *IGF1*, *ERBB2* in osteoporosis or osteogenesis [7, 17–24]. For example, Liu et al. reported that p53 inhibits osteogenesis by affecting the function of MSCs via miRNA signaling pathways [20]. Xie et al. found that *CTNNB1* and *TP53* are the most upregulated DEGs and may play a crucial role in primary osteoporosis [7]. It was also reported by that signaling in the MAP kinase pathway is differentially activated in MSCs derived from osteoporotic postmenopausal women [17], and that miR-378 overexpression attenuates high glucose-mediated suppression of osteogenic differentiation by targeting CASP3 and activating the PI3K/Akt pathway [23]. In addition, Notch appears to suppresses osteoblast differentiation, at least in part by restricting glucose metabolism [18], and inhibiting microRNA-139-5p promotes osteogenic differentiation of BMSCs by activating the Wnt/beta-catenin signaling pathway by targeting NOTCH1 [21]. CDK1, which is essential for osteoblast proliferation, functions as a molecular switch that shifts osteoblast proliferation to maturation [22]. IGF-1 is a key regulator of longitudinal skeletal growth and is an anabolic hormone that influences bone modeling and remodeling throughout life [19]. Finally, sustained activation of ErbB1 and ErbB2 enhances hMSC osteogenesis [24]. All these findings are consistent with the bioinformatics analysis reported in the present study.

P53 had the highest degree score in the PPI network, indicating it may play a central role during the development of osteoporosis. P53 is encoded by the tumor suppressor gene *TP53* and suppresses tumor growth by slowing cell growth and division. In the present study, it is found that serum p53 levels are increased in osteoporosis patients, and knocking down p53 partially reversed decreases in BMD in vitro and in vivo. In addition, GO and KEGG enrichment analyses indicate that p53 is involved in “the cancer pathway,” “proteoglycans in cancer pathway,” and “P53 signaling pathway.” We therefore suggest that p53 may contribute to the pathogenesis of osteoporosis via these pathways.

There are a few limitations to this study. First, only one dataset was found for the bioinformatics analysis. Analysis of additional datasets would strengthen the evidence from bioinformatics analyses. Second, we did not perform a subgroup analysis of different osteoporosis subtypes, which may involve different signaling pathways and genes. A future study will focus on investigating how p53 interacts with other osteoporosis-related pathogenic genes in different osteoporosis subtypes.

In summary, our findings suggest that p53 may play a key role in the development of osteoporosis, and that suppressing some of the activities of p53 may inhibit the development and/or progression of osteoporosis. For that reason, we suggest p53 should be considered a potential therapeutic target for the treatment of osteoporosis.

## MATERIALS AND METHODS

### Search of microarray data and identification of DEGs

The microarray dataset GSE100609 for osteoporosis patients and control subjects were retrieved from the NCBI Gene Expression Omnibus (GEO). The expression data were then uploaded to and analyzed by Morpheus (Morpheus, https://software.broadinstitute.org/morpheus), a versatile online matrix visualization and analysis software package. The gene expression was visualized using a heatmap. Signal to noise >1 or signal to noise <1 is considered as DEGs.

### GO and KEGG pathway enrichment analysis

The DAVID version 6.8 online bioinformatics resources tool was used to perform GO term and KEGG pathway enrichment analyses [25, 26].

### PPI network analysis

The PPI network was used to further explore the functional interactions between DEGs imported into Search Tool for the Retrieval of Interacting Genes (STRING, https://www.string-db.org). Interactions with a combined score >0.5 were identified. The PPI network was then built using Cytoscape software (version 3.7.2). Modules of the PPI networks with a Molecular Complex Detection (MCODE) score ≥5 and >5 nodes were identified using the MCODE plugin in Cytoscape. Function enrichment analysis of DEGs in the top module was performed with DAVID. The DEGs were then ranked based on degree centrality using the Centiscape 2.2 plugin in Cytoscape.

### Blood collection

Between May 2016 and June 2018, peripheral blood samples were collected from patients in Shanghai Tongji Hospital (10 healthy volunteers, 10 osteoporosis patients) for gene expression analysis. The patient studies were approved by the Committees of Clinical Ethics in the Tongji Hospital (Tongji University of Medicine, Shanghai, China), and informed consent was obtained from all participants.

### Animals and specimen collection

Thirty female C57BL/6 mice (6 weeks old) were obtained from the Department of Animal Science of Tongji Hospital, Tongji University School of Medicine. A mouse osteoporosis model is established using oophorectomy. Following oophorectomy, the 30 mice were randomly divided into three groups: a control group treated with PBS (n=10), a siRNA-NC group treated with negative control siRNA (n=10), and a siRNA-p53 group treated with p53 siRNA (n=10). We administered subcutaneously injected 5 ml of solution per kilogram body weight every 2 days for 10 weeks. The weights of the animals were recorded weekly during the experimental period. All animal experiments adhered to the National Institutes of Health Guide for the Care and Use of Laboratory Animals and were performed according to the protocols approved by the Medical Research Ethics Committee of the Tongji Hospital, Tongji University of Medicine.

### Cell culture and transfection

hMSCs were kindly donated by the Tongji Hospital, Tongji University of Medicine, Shanghai, China. Cells were maintained for a maximum of 4 passages in specific medium designed for human mesenchymal stem cells at 37°C in a 5% CO_2_ incubator. Cells were transfection with 50 nM siRNA-NC or siRNA-p53 (GenePharma, Shanghai, China) using Lipofectamine 3000 (ThermoFisher Scientific, MA, USA, #L3000001) following the manufacturers protocol.

### qRT-PCR analysis

qRT-PCR analysis was performed as described previously [27]. Briefly, Trizol reagent (ThermoFisher Scientific, MA, USA, #L15596026) was applied to extract total RNA. cDNA was then synthesized using a qPCR RT Master Mix kit (Toyobo, Osaka, Japan). Relative expression levels of mRNA were calculated using the 2-ΔΔCt method (Ct of GAPDH minus the Ct of the target genes) and normalized to the level of U6 mRNA determined as the 2-ΔΔCt. The primer sequences used were as follows: for p53, CAGCACATGACGGAGGTTGT (forward) and TCATCCAAATACTCCACACGC (reverse); for Col 1a1, TGGCAAAGATGGACTCAACG (forward) and TCACGGTCACGAACCACATT (reverse); for ALP, GCTCTGGAAAGTCCTTCAAAGC (forward) and TCTTCTTCCCTGGACACTGCC (reverse); for OCN, TCACACTCCTCGCCCTATTG (forward) and CTCCTGAAAGCCGATGTGGT (reverse); for Runx2, CTACTATGGCACTTCGTCAGGAT (forward) and ATCAGCGTCAACACCATCATT (reverse); and for GADPH, GGAAGCTTGTCATCAATGGAAATC (forward) and TGATGACCCTTTTGGCTCCC (reverse).

### Western blotting analysis

Cell lysates were prepared using NETN buffer (20 mM Tris HCl [pH 8.0], 100 mM NaCl, 1 mM EDTA and 0.5% Nonidet P-40) and were resolved by SDS-PAGE. Proteins were transferred to PVDF membranes, which were blocked in 5% skim milk overnight at 4°C. The membranes were probed with primary antibodies and labeled with HRP-conjugated secondary antibodies (Aspen, Johannesburg, South Africa, #AS1058). Chemiluminescence detection systems (Canon, Tokyo, Japan, #LiDE110) were used to visualize the protein bands. The antibodies used were as follows: anti-collagen I (1:500, Abcam, MA, USA, #ab34710), anti-ALP (1:1,000, Abcam, MA, USA, #ab95462), anti-Osteocalcin (1:500, Abcam, MA, USA, #ab93876), anti-RunX2 (1:500, Abcam, MA, USA, #ab23981), anti-p53 (1:5,00, #ab131442, Abcam, MA, USA), and anti-GAPDH (1:10,000, Abcam, MA, USA, #ab37168).

### Micro-CT analysis

All samples were scanned using a BRUKER SkyScan 1276 scanner microCT system (BRUKER, Karlsruhe, Germany) to provide images at 2400 views, 5 frames/view, 37 kV, and 121mA. The bone trabeculae selected were approximately 0.2 mm below the proximal humerus growth plate and measured 0.5 mm in length. After scanning, calluses were preserved at -80°C for miRNA extraction, qRT-PCR, and western blot analysis. Measurement parameters were as follows: bone volume (BV), total volume (TV), BV/TV, and BMD.

### Statistical analysis

GraphPad Prism 8.0 (GraphPad Software, Inc.) was used for statistical analyses. The data are presented as the mean ± standard deviation (SD). Comparisons between two groups were made using Student’s t-test. Comparisons among three groups were made using one-way ANOVA with post-hoc Tukey’s test.
